# Calories and sugars in boba milk tea: implications for obesity risk in Asian Pacific Islanders

**DOI:** 10.1002/fsn3.362

**Published:** 2016-03-29

**Authors:** Jae Eun Min, David B. Green, Loan Kim

**Affiliations:** ^1^ Department of Nutritional SciencesPepperdine University24255 Pacific Coast HighwayMalibuCalifornia90263; ^2^ Department of ChemistryPepperdine University24255 Pacific Coast HighwayMalibuCalifornia90263

**Keywords:** Asian Pacific Islanders, boba, calorific value, obesity, saccharides, sugar‐sweetened beverages

## Abstract

In the last several decades, obesity rates have reached epidemic proportions, and increases the risk for a host of comorbidities, including diabetes, cardiovascular disease, and certain kinds of cancers. Boba milk tea, first became popular in the 1990s throughout Asia, and has gained more popularity in the United States and in Europe since 2000. Currently, available nutrition data from online sites suggest this beverage contains high amounts of sugar and fat. One published nutrition study suggests that boba tea drinks are part of the larger group of sugar‐sweetened beverages (SSB) because these beverages are usually sweetened with high‐fructose corn syrup (HFCS). This study experimentally determined the sugar composition (sucrose, fructose, glucose, and melezitose) and calorific values of boba milk tea drinks and their components. Results suggested that boba drinks fit the US Dietary Guidelines definition of a SSB. One 16‐ounce boba drink exceeds the upper limit of added sugar intake recommended by the 2015 US Dietary Guidelines Advisory Committee. The high caloric and sugar content of boba beverages pose public health concerns as they have the potential to further exacerbate the childhood obesity epidemic. Nutrition education targeting Asian populations should give special attention to boba tea as a SSB. Also, prudent public health recommendations should be suggested for moderate consumption of these beverages. With the growing popularity of boba beverages in the United States, the findings from this study provide public health practitioners with valuable data on how boba beverages compare with other SSBs.

## Introduction

Boba milk tea, also known as bubble or pearl tea, first became popular in the 1990s throughout Asia, and has gained more popularity in the United States and in Europe since 2000 (Alexander 2001). The origin of this beverage dates back to the 1980s in Taiwan, where a Taiwanese tea shop owner, Liu Han‐Chieh, and his product development manager, Lin Hsiu Hui, experimented with adding different ingredients such as fruit, syrup, and Tapioca ball in milk tea beverages (Chang 2012). The central ingredient in this type of drink is “boba” or “pearl” balls. These boba balls are made of tapioca, which are boiled to produce a round chewy ball that is then added to hot and cold beverages such as tea, coffee, smoothies, slushies, and blended beverages. These beverages are usually consumed with a large straw, through which the tapioca balls are slurped and chewed.

Boba tea beverages are especially popular in large metropolitan areas with high concentrations of Asian American Pacific Islander (AAPI) youth and young adults (Rosen 2015). Evidence of this drink's popularity in the United States is clear: a quick search on Yelp using the key words “boba tea” yields more than 200 listings of boba tea stores, just in the Los Angeles area alone (Yelp, 2016). The popularity of boba tea has expanded to Europe, with boba stores now in metropolitan cities such as London (Townsend 2014). In the United States, the popularity of boba is such that there is a designated page on Wikipedia to detail the history of boba tea, along with a listing of over 13 boba store chains, and descriptions of over 200 variations in boba drinks (Tea 2016, Wikipedia, 2016). As it has become so popular and commonplace, individuals can purchase their own boba ingredients and watch YouTube videos to learn techniques for how to make boba teas at home (Nuts, 2016). There is even a boba school devoted to teaching the trade of making boba drinks to clients interested in opening a boba store (Wei 2015).

Over the years, the original boba milk tea drink has expanded to consist of more variations and combinations. Internet blogs describe boba as more than just tea drinks; some have suggested that these are tea desserts (Rosen 2015). The primary component in boba teas are the black tapioca “boba” balls, which are usually made from cassava starch, sweet potato, and brown sugar. Less commonly, white tapioca pearls are used, and these are made from cassava starch, caramel, and chamomile root and have a different flavor (Weil 2012). In addition, other ingredients that are commonly added to boba drinks include jelly (Nata de coco) and egg pudding. Additionally, other variations in boba drinks include fruit smoothies and slushies, blended coffee and tea combinations.

Currently, available nutrition data on boba drinks are from popular press or online sources, but there's a paucity of published research to document the nutritional quality of these beverages. Online nutrition facts data from large boba chains suggest this beverage contains high amounts of sugar and fat (Weil 2012). A search on the internet for calorific value of boba drinks found that a 16‐ounce serving of boba milk tea contains between 200–450 calories, depending on the type of boba beverage and what additional ingredients are included (FatStraws, 2015; Weil 2012; Carey 2015). One published nutrition study conducted by Taiwanese researchers Chan and colleagues suggests that boba tea drinks are part of the larger group of sugar‐sweetened beverages (SSB) because these beverages are usually sweetened with high‐fructose corn syrup (HFCS; mainly HFCS‐55) (Chan et al. 2014). Other than this study and a plethora of online articles about the nutritional quality of boba tea drinks, there is still a lack of knowledge about the calorific values or sugar source of these boba drinks. Therefore, the purpose of this study was to analyze the sugar composition (sucrose, fructose, glucose, and melezitose) and calorific value of boba milk tea drinks. The findings of this study have important implications for public health as knowledge of the nutritional composition of boba drinks will allow public health researchers to evaluate whether this beverage should be classified as a SSB, and if so, how these beverages influence health and obesity in the United States

## Material and Methods

### Reagents

Boba milk tea and the added ingredients (egg pudding, jelly, tapioca “boba” balls) were purchased at a local boba chain store located in a densely populated Asian community in Los Angeles, California. The milk tea boba and components purchased were the most typical boba drink as described by the proprietor and came in the standard size (473 mL or 16 ounces). The added ingredients of tapioca “boba” balls, egg pudding, and jelly were chosen because they represented the common add‐on ingredients in boba drinks. Three samples each of equivalent lots of each component were purchased. After purchase, drinks or individual components were refrigerated in preparation for analyses. Sugar standards, acetic acid, and benzoic acid (Fisher Scientific, Hanover Park, IL, USA) were ACS grade or better and used without further purification. Water was deionized by ion exchange to a resistivity >16 MΩ‐cm and filtered to 0.2 *μ*m (Barnstead Nanopure II).

Solid‐phase extraction cartridges were purchased from Phenomenex (Torrance, CA). For reversed‐phase extractions, 3‐ mL cartridges packed with 500 mg of Strata C18‐E (55 *μ*m, 70Å) sorbant were utilized. For ion‐exchange extractions, 6‐ mL cartridges packed with 1 g of Strata ABW (55 *μ*m, 70Å) mixed‐bed ion‐exchange resin were used.

### Sample preparation for saccharides determinations

Samples were prepared using a published method adapted to the liquid, solid, and semisolid components of a boba beverage (Brereton and Green 2012). All the boba beverage components were refrigerated at 4°C and were brought to room temperature prior to use. Liquid samples by diluting 100‐ *μ*L aliquots with 1 mL of 10 mmol/L acetic acid in 1.5‐ mL microcentrifuge tubes, followed by vortexing to completely mix the suspension. Semisolid samples (egg pudding) were prepared by combining 100 mg of the foodstuff with 1 mL of 10 mmol/L acetic acid in 1.5‐mL microcentrifuge tubes followed by vortexing to disperse and suspend the viscous pudding. Solid foodstuffs (tapioca and jelly) were prepared by weighing 100 mg (wet mass) of a foodstuff into a 1 mL Dounce tissue grinder (Wheaton Science, Millville, NJ, USA) and macerating with 1 mL of 10 mmol/L acetic acid. The suspension was transferred to a microcentrifuge tube and the tissue grinder was rinsed three times with 10‐mmol/L acetic acid. Each vial was centrifuged (Eppendorf Microcentrifuge, Hauppauge, NY, USA) for 5 min at 16,000 × *g*.

Triglycerides, fatty acids, and lipids remaining in the supernatant of the centrifuged samples were removed by reversed‐phase solid‐phase extraction. A Strata C18‐E cartridge was preconditioned by flushing with 2 mL of methanol followed by 3 mL of water at a flow rate of 4 mL/min. The supernatant was extracted at a flow rate of 3–4 mL/min and the eluent collected into a polyethylene tube. The centrifuge vial was rinsed twice with 1 mL of deionized water, and each rinse was subsequently used to wash the cartridge bed and was combined with the extracted solution.

Any remaining salts and proteins were removed from the sample by mixed‐bed ion exchange. A Strata ABW mixed‐bed ion‐exchange cartridge was conditioned by flushing with 2 mL of methanol followed by 3 mL of water at a flow rate of 4 mL/min. The entire eluent obtained from the reversed‐phase extraction was extracted with the ABW cartridge with an additional 1 mL of water to rinse the vial and flush the ABW cartridge. The extract was diluted to a standard volume and immediately analyzed by high‐performance liquid chromatography or stored at 4°C for later analysis. All samples were analyzed within 7 days of preparation. Triplicate preparations were performed for all samples.

### High‐performance liquid chromatographic analysis

Saccharide analysis was performed by ligand‐exchange chromatography using a Rezex RCM Monosaccharide column (Ca^2+^‐loaded, 25 cm × 7.8 mm, 8 *μ*m *d*_p_, Phenomenex, Inc.) protected with a SecureGuard^®^ guard cartridge (Phenomenex) coupled to a Spectra Physics SP8800 HPLC pump and Thermo Separations RefractomonitorIV refractive index detector. The column was thermostatted at 85°C. The pure water mobile phase was vacuum degassed throughout the analysis and the flow rate was 0.6 mL/min. The manual injection volume was 20 *μ*L and samples were filtered through a 4‐mm‐diameter 0.2 *μ*m PVDF membrane syringe‐tip filter (Millipore, Billerica, MA, USA) during the injector load step. The identity of the saccharides was determined by comparison to authentic standards and quantitation was by comparison of integrated peak areas to rectilinear calibration plots.

### Determination of calorific values by bomb calorimetry

A measured volume of liquid samples was transferred to an aluminum evaporating dish and dried to constant mass at 80°C (~48 h). Solid and semisolid samples were macerated with an equal mass of water to a homogeneous suspension in a blender (Waring Conair Corp., South Shelton, CT, USA). Slurries were transferred to aluminum evaporating dishes and dried to constant mass at 80°C (~48 h). Weighed portions of the dried samples were ground with an equal mass of benzoic acid, and ~1‐g portions were pressed into 13‐mm‐diameter pellets for bomb calorimetric analysis.

Bomb calorimetry was performed with a Parr Plain Jacket Oxygen Bomb Calorimeter (Model 1341) equipped with a digital thermometer (Parr Model 6775). Two liters of deionized water was placed in the static jacket for every measurement and allowed to thermally equilibrate. Constant initial temperature was assumed when the temperature was stable for longer than 30 sec. Temperatures were measured to a precision of ±0.001°C. The calorimeter was calibrated with benzoic acid. Standard and sample masses were nominally 1 g. The oxygen bomb was purged and charged to 30 bar with oxygen. Three replicates of standards and every sample were performed.

## Results and Discussion

**Figure 1:**
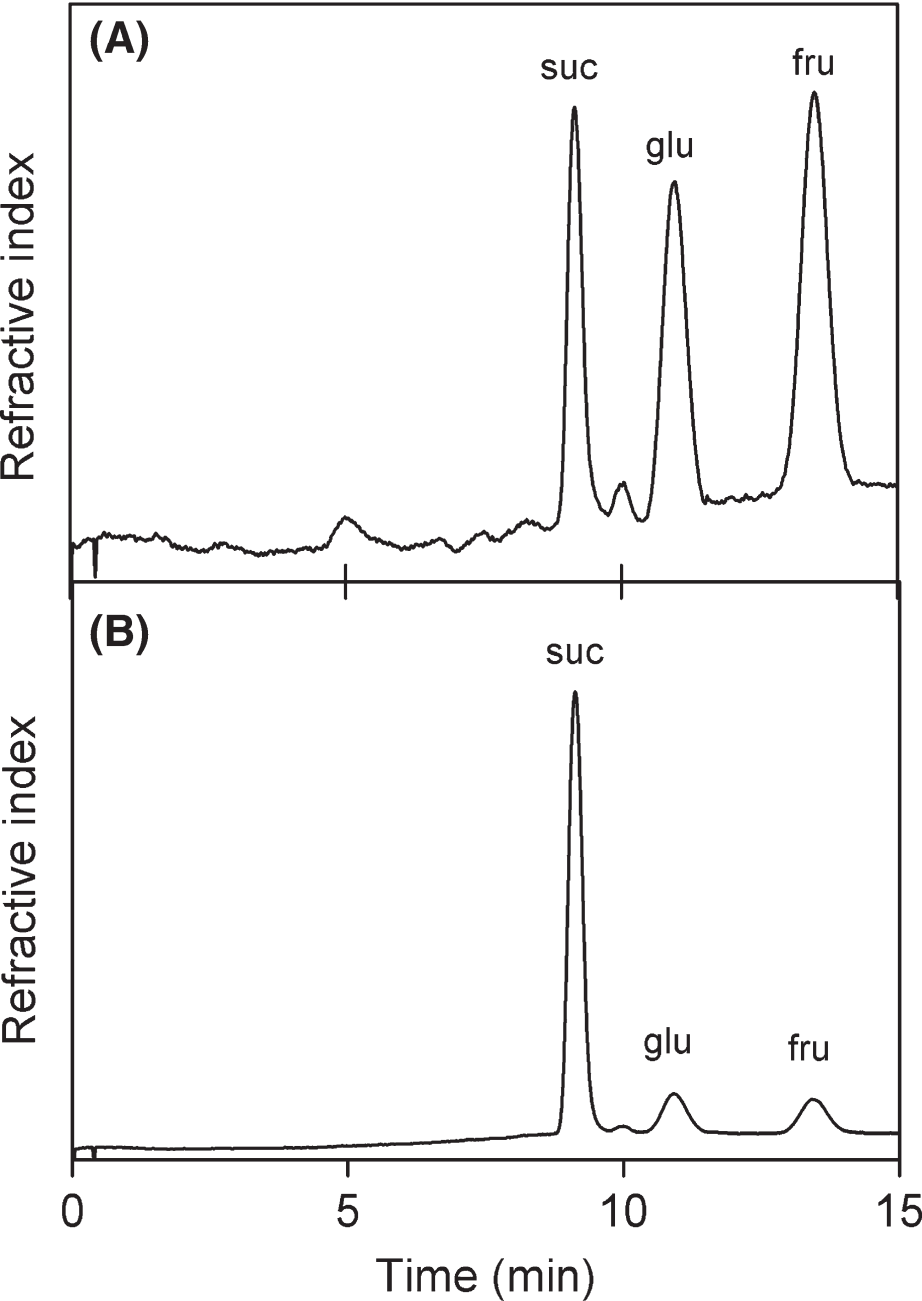
Representative chromatograms of (A) tapioca and (B) jelly (Nate de Coco).

Figure 1 shows representative chromatograms for saccharides extracted from the tapioca balls and the jelly (Nata de Coco). The large peak area for the fructose in Figure 1A strongly suggests that high‐fructose corn syrup was the sweetener in the pudding. The jelly (Fig. 1B) is sweetened with cane or beet sugar, as demonstrated by the sucrose band dominating the chromatogram. According to the 2010 Dietary Guidelines for Americans, sugar‐sweetened beverages (SSBs) are defined as, “liquids that are sweetened with various forms of sugars that add calories. These beverages include, but are not limited to, soda, fruit‐ades and fruit drinks, and sports and energy drinks” (USDA, 2010). Based on the experimentally determined sugar composition presented in Figure 1, the boba milk tea beverage fits the US Dietary Guidelines definition of a sugar‐sweetened beverage.

Table 1 summarizes the amounts of each detected saccharide for every ingredient in a boba beverage, with reported values as averages of triplicate measurements. The assays in Table 1 are scaled and reported for a typical serving size of 473 mL (16 US oz) of milk tea, 60 g of tapioca, 50 g of jelly, and 80 g of egg pudding. The calorific values are reported in food calories (kcal) and represent the pooled energies from protein, fat, and saccharides for each component, as determined by bomb calorimetrically. Calorific values may slightly overestimate metabolizable energy due to the inclusion of insoluble fiber and unavailable carbohydrates in the measurements (Maynard 1944; Livesey 2002; Zou et al. 2007; Trivedi 2009). Table 2 reports the total saccharide and calorific values for different combinations of boba beverage ingredients. Results indicate a 16 ounce (473 mL) single serving of a boba drink with milk tea and tapioca “boba” balls containing 299 calories and 38 grams of sugar.

**Table 1:** Summary of saccharide concentrations and calorific values for each component of a boba beverage. Values have also been scaled to one serving size. Calorific values are pooled: starch, protein, and fat were not measured individually

Component	Assay^a^		1 Serving size^b^	
	Sugar (mg/g)	Calorific value (kcal/g)	Sugar (g)	Calorific value (kcal)
Milk Tea^c^		0.53 (0.07)		262.6 (35.4)
Melezitose	3.50 (0.06)		1.65 (0.03)	
Sucrose	2.65 (0.48)		1.25 (0.23)	
Glucose	29.49 (0.34)		13.95 (0.16)	
Fructose	43.95 (0.28)		20.79 (0.13)	
Total	79.59 (0.95)		37.65 (0.45)	
Tapioca		1.30 (0.01)		77.9 (0.7)
Sucrose	19.76 (0.87)		1.19 (0.05)	
Glucose	32.21 (0.63)		1.93 (0.04)	
Fructose	56.8 (1.2)		3.41 (0.07)	
Total	108.8 (2.6)		6.53 (0.16)	
Jelly		4.24 (0.04)		212.2 (2.1)
Sucrose	171.5 (1.5)		8.58 (0.08)	
Glucose	24.42 (0.05)		1.22 (0.00)	
Fructose	36.39 (0.35)		1.82 (0.02)	
Total	232.3 (1.1)		11.62 (0.06)	
Egg pudding		0.67 (0.004)		53.7 (0.3)
Melezitose	2.74 (0.11)		0.22 (0.01)	
Sucrose	1.35 (0.72)		0.11 (0.06)	
Glucose	114.2 (1.5)		9.14 (0.12)	
Fructose	107.1 (9.1)		8.57 (0.73)	
Total	225.4 (9.8)		18.03 (0.79)	

a Values in parentheses are 1 standard error (*n *=* *3).

b Milk tea: 473 mL (16 US oz); Tapioca: 60 g; Jelly: 50 g; Egg Pudding: 80 g.

c Sugar concentration is reported in mg/mL and calorific value is reported in Cal/mL.

**Table 2:** Sugar and calorific values in boba beverage served with differing combined components. Small cup = 473 (16 oz); Large cup = 946 mL (32 oz)

Small cup	Sugar (g)	Calorific value (kcal)	Large cup	Sugar (g)	Calorific value (kcal)
MT + TAP	38	299	MT + TAP	57	448
MT + J	43	269	MT + J	72	431
MT + EP	49	275	MT + EP	75	398
MT + TAP + J	42	292	MT + TAP + J	74	493
MT + TAP + EP	48	297	MT + TAP + EP	77	459
MT + J + EP	53	267	MT + J + EP	93	444
MT + J + EP + TAP	57	323	MT + J + EP + TAP	96	515

Reported values have been corrected for changes in serving sizes when components are mixed. MT, milk tea; TAP, tapioca; J, jelly; EP, egg pudding.

The 2010 Dietary Guidelines recommend that no more than about 5–15% of the total daily calories come from added sugar and solid fats in the diet (DGAC, 2010), whereas the American Heart Association suggests Americans to limit added sugars to not more than 150 kcal/day for men and 100 kcal/day for women (Johnson et al. 2009; AHA, 2015). After applying the Atwood factor (Maynard 1944; Zou et al. 2007) to the pooled saccharide measurements, the amount of calories and added sugar in this boba milk tea beverage contains the entire quantity of the recommended maximum daily intake of sugar for men (38 g) (DGAC, 2010) and over 150% for women (25 g) (Johnson, 2009). When combined with the additional ingredients in the beverage, the pooled saccharides easily exceed the recommended maximum daily intake of sugar for all populations. A large 32 US oz (946 mL) serving of boba milk tea that includes jelly and egg pudding supplies more than 250% and 384% of the recommended maximum daily intake of sugar for men and women, respectively.

The concern over SSBs is derived from mounting evidence which links intakes of added sugars from SSBs with increased body weight, type 2 diabetes, metabolic disease, and a host of other obesity‐related comorbidities (Malik et al. 2010; Bray and Popkin 2014). As a result, the most recent 2015 Dietary Guidelines Advisory Committee (DGAC) issued even stronger and more specific guidelines with regards to added sugar. In the report, DGAC suggests limiting added sugars intake to below 10% of total energy intake (USDA, 2015). Based on a 2000 calorie diet, this equates to not more than 200 calories per day (50 grams or 12.5 teaspoons of sugar). The findings suggest that one 16‐ounce boba beverage with just milk tea and boba easily exceeds the upper limits of these most recent DGAC recommendations. As seen in Table 2, this is a “basic” boba beverage; other added ingredients that accompany this beverage, such as jelly and egg pudding, can result in total calories well above 16% of total energy intakes; a larger size boba beverage with all the ingredients exceeds 500 calories, and contributes to 25% of total daily calories. As evident from Table 3, most of the different categories of SSB, including boba drinks, exceed these guidelines, and thus increase individuals' risk of obesity.

**Table 3:** A comparison of sugar content and calorific values in some popular sugar‐sweetened beverages

Beverage	1 Serving size (16 oz)	
	Sugar (g)	Calorific value (kcal)
Milk tea	38	263
Milk tea w/ Tapioca	38	299
Cranberry juice cocktail	67	267
Orange soda	62	227
Energy drink	62	240
Orange juice	56	227
Cola	56	200
Sweetened ice tea	44	168
Sports drink	28	120

Sugar and calorific values were derived from: http://cdn1.sph.harvard.edu/wp-content/uploads/sites/30/2012/10/how-sweet-is-it-color.pdf.

In the last several decades, obesity rates (defined as a body mass index (BMI) range between 25–29.9 kg/m^2^, whereas obesity is having a BMI of 30 kg/m^2^ or higher) have reached epidemic proportions, with two of every three adults and one of three children being either overweight or obese (Flegal et al. 2012). Childhood obesity (defined as above the 95^th^ percentile based on CDC age‐ and gender‐specific growth charts) has more than doubled in children and quadrupled in adolescents. The percentage of children aged 6–11 years in the United States who were obese more than doubled, from 7% in 1980 to nearly 18% in 2012. Similarly, the percentage of adolescents aged 12–19 years who were obese quadrupled, from 5% to nearly 21% over the same period. (CDC, 2015a) More than one third of children and adolescents were overweight or obese in 2012 (CDC, 2015c). Obesity results in a host of comorbidities such as diabetes, cardiovascular disease, and certain kinds of cancers (CDC, 2015b). Children who are obese during adolescence are more likely to struggle with obesity and other chronic diseases during their adult years, ultimately resulting in a shortened life span. Treating obesity‐related health conditions has been estimated to cost $190 billion a year for the nation (Cawley and Meyerhoefer 2012).

Among adolescents, increasing attention has been focused on addressing the consumption of SSB as a risk factor for obesity. It is well documented in the health literature that SSB contributes a significant amount of sugar, total calories, and has been suggested to result in higher rates of obesity, cardiovascular disease, diabetes, and gout (Swinburn et al. 2004; James and Kerr 2005; Hu and Malik 2010; Harvard, 2012). The most common types of SSB in the current US food supply are sodas, juices, and energy drinks. This study establishes boba beverages as yet another type of SSB, and these results have important public health implications with regards to the obesity epidemic. Sugar‐sweetened beverages do not displace calories from food; instead, they provide “add‐on” calories and thus increase total caloric intake, resulting in weight gain over time, and thus higher obesity risk. The high dietary glycemic load from SSBs due to fructose, in the form of HFCS or sucrose, can increase the risk for hepatic insulin resistance, visceral fat deposition, and elevated triglyercerides and cholesterol (Bray and Popkin 2014). Furthermore, recent research suggests that drinking about 1 L or the equivalent of two 16 ounce SSBs per day for 6 months can induce features of metabolic syndrome and fatty liver (Bray and Popkin 2014).

Given that boba drinks have similar calories and sugar content as other SSBs, this is a significant public health concern, particularly among Asian youth who may be consuming these boa tea beverages daily, in addition to other SSBs such as sodas and energy drinks. Although there are no available published data on boba consumption among youth in California, it is estimated that about half of children, teens, and adults drink at least one serving of SSB daily (Keihner et al. 2012). Overconsumption of these SSBs and boba tea can likely contribute to higher rates of overweight among Asian youth, further contributing to the current obesity epidemic.

The sugar and total caloric content of boba drinks is particularly concerning with regards to obesity rates in Asian youth as research suggests that “Asian countries have a low prevalence of obesity, yet high rates of obesity‐related diseases.”(Choo 2002) A study conducted by Chiu and colleagues found that South Asians and Chinese develop diabetes at lower ranges of BMI than their white counterparts (Chiu et al. 2011). Other studies suggest that Asian populations' BMI cutoff point to be lower than the international BMI cutoff point that was recommended by World Health Organization (WHO) (Low et al. 2009). Asians have different body build and muscularity than the white population, leading Asians to have a lower BMI (by about 2–3 kg/m^2^) (Deurenberg et al. 2002). Therefore, WHO proposed lowering BMI cutoff point to 23–27.5 kg/m^2^ as overweight and ≥27.5 kg/m^2^ as obese (Shiwaku et al. 2004). Jih and colleagues investigated the prevalence of overweight and obesity of AAPI in California using the lower BMI and found there was an increase number of AAPI overweight and obese that met the new WHO Asian BMI criteria (Jih et al. 2014). As the Asian BMI criteria are not currently used in national‐ and state‐wide health surveys, it is likely that the Asian obesity rates are underreported, and thus at greater risk for obesity‐related chronic diseases.

Therefore, prudent public health recommendations suggest moderate consumption of these beverages. Additionally, healthier alternatives are advised when ordering boba drinks, such as choosing boba tea without the milk, requesting lower sugar options (half sugar or one‐third sugar are available), or leaving out additional ingredients such as pudding, jelly, and tapioca. These alternatives can reduce the overall caloric value of the boba tea beverages, thus fitting better within the Dietary Guidelines for added sugars.

There are several limitations to this study. First, boba beverages were purchased from one retail boba store, and thus our analysis cannot be generalized to all boba beverages. As each boba store uses different ingredients and different methods for making boba drinks, this adds to the challenge of being able to generalize these findings to all boba drinks. Additionally, the scope of our study was limited to analysis of boba milk tea with a few additional ingredients. We did not analyze other types of hand‐shaken boba beverages, such as tea‐only drinks, smoothies with boba, or juice boba drinks. Also, while these boba beverages contain tea, which have potentially beneficial antioxidants, we did not analyze the composition of tea in boba drinks, and therefore cannot draw any conclusions regarding the potential antioxidant benefits of boba beverages. Finally, our analysis was limited to determining total calories and carbohydrate composition in the boba milk tea beverages. Future studies should examine the composition of protein, fat, and antioxidants in boba beverages. Despite the limitations of this study, this is the first work to document the calorific value and sugar composition of boba beverages. These findings advance the nutrition literature and are an important first step in understanding how this beverage fits in the landscape of sugar‐sweetened beverages in the United States

## Conclusion

This study is the first of its kind to document experimentally derived caloric value and sugar composition in boba milk tea. Our findings suggest that boba drinks fit the US Dietary Guidelines definition as a sugar‐sweetened beverage. Additional, one 16‐ounce boba drink exceeds the upper limit of added sugar intake recommended by the 2015 US DGAC. Given this important knowledge about the caloric value and sugar source of boba milk tea drinks, nutrition education targeting Asian populations should give special attention to boba tea as a SSB. Given that this popular beverage will only become more popular, recommendations from public health practitioners working in Asian communities should suggest moderate consumption of boba drinks, choosing options for less sugar, and not adding other ingredients such as pudding or jelly. As this work is the first to document the nutritional value of boba beverages, research on health risks associated with drinking boba beverages warrants further study. As our study was unable to determine how often boba tea can be consumed without increasing risk of obesity, this is also an important area for future research. With the growing popularity of boba beverages in the United States and around the world, the finding from this study is an important step toward understanding how boba beverages compare to other SSBs.

## Conflict of Interest

None declared.
